# A Comparison of Existing Bootstrap Algorithms for Multi-Stage Sampling Designs

**DOI:** 10.3390/stats5020031

**Published:** 2022-06-06

**Authors:** Sixia Chen, David Haziza, Zeinab Mashreghi

**Affiliations:** 1 Department of Biostatistics and Epidemiology, University of Oklahoma Health Sciences Center, Oklahoma City, OK 73104, USA;; 2 Department of Mathematics and Statistics, University of Ottawa, Ottawa, ON K1N 6N5, Canada; 3 Department of Mathematics and Statistics, University of Winnipeg, Winnipeg, MB R3B 2E9, Canada;

**Keywords:** bootstrap algorithms, multi-stage sampling, Taylor linearization, variance estimation

## Abstract

Multi-stage sampling designs are often used in household surveys because a sampling frame of elements may not be available or for cost considerations when data collection involves face-to-face interviews. In this context, variance estimation is a complex task as it relies on the availability of second-order inclusion probabilities at each stage. To cope with this issue, several bootstrap algorithms have been proposed in the literature in the context of a two-stage sampling design. In this paper, we describe some of these algorithms and compare them empirically in terms of bias, stability, and coverage probability.

## 1. Introduction

Many surveys conducted by national statistical offices use stratified multi-stage sampling designs for selecting a sample. Reasons for using multi-stage sampling rather than direct element sampling include the lack of element-level sampling frames and cost considerations when data collection involves face-to-face interviews. Stratified multi-stage sampling designs include some form of stratification, selection of primary sampling units (psu), and subsampling within selected psus. This is especially common in social and health surveys. For general multi-stage sampling designs, unbiased variance estimation is a complex task as it relies on the availability of the second-order inclusion probabilities at each stage. If the first-stage sampling fraction is small, a common variance estimation strategy is to pretend that the psus were selected with replacement and use the customary with replacement variance estimator. The resulting estimator is generally conservative in the sense that it may suffer from a small positive bias.

Another approach to variance estimation for survey data is bootstrap variance estimation originally proposed by Efron [1] in the context of independent and identically distributed observations. In a finite population sampling, bootstrap procedures can be classified into two broad groups. In the first, bootstrap samples are selected from the original sample; e.g., [2,3] among others. Rao and Wu [2] applied a scale adjustment directly to the survey data values so as to recover the usual variance formulae. Rao *et al.* [4] presented a modification of the method of Rao and Wu [2], where the scale adjustment is applied to the survey weights rather than to the data values. The second group of procedures consists of first creating a pseudo-population from the original sample. Bootstrap samples are then selected from the pseudo-population using the same sampling design utilized to select the original samples; see [5–8], among others. Many of the aforementioned bootstrap procedures may be implemented by randomly generating bootstrap weights so that the first two (or more) design moments of the sampling error are tracked by the corresponding bootstrap moments; see [9,10]. These procedures are often referred to as bootstrap weight procedures. For a comprehensive review of bootstrap procedures for survey data, the reader is referred to Mashreghi *et al.* [11].

The goal of this paper is to empirically compare several existing bootstrap algorithms that have been proposed in the literature for two-stage sampling designs. The bootstrap procedures are compared with respect to bias, stability, and coverage probability of confidence intervals. In Section 2 we present the basic setup and discuss some classical variance estimation procedures for two-stage sampling designs. In Section 3, we present some bootstrap algorithms proposed in the case of simple random sampling without replacement in both stages. Bootstrap algorithms for unequal probability sampling designs are described in Section 4. In Section 5, we present the results from a simulation study. We make some final remarks in Section 6.

## 2. The Setup

Consider a finite population 𝒰 consisting of N primary sampling units (psu), ${𝒰}_{1},\ldots ,{𝒰}_{N}$, of size $M_{1},\ldots ,M_{N}$, such that $𝒰={⋃}_{i\in U}\;{𝒰}_{i}$ and ${𝒰}_{i}\cap {𝒰}_{j}=\emptyset$ if i≠j. Let $M_{0}=\sum_{i\in U}\;M_{i}$ be the total number of elements in the population. We are interested in estimating a population total $t_{y}$ of a survey variable y:

$$t_{y}=\sum_{i\in 𝒰}\;t_{i},$$

where $t_{i}=\sum_{k\in {𝒰}_{i}}\;y_{ik}$ denotes the ith psu total, i=1,…,N, and $y_{ik}$ denotes the y-value for the kth element in the ith psu. To that end, we select a sample according to a two-stage sampling design:

- A sample $S_{1st}$ of psus, of size n, is selected according to a given sampling design $P\left(S_{1st}\right)$ with first-order inclusion probabilities, $\pi_{i}=P\left(i\in S_{1st}\right)$, and with second-order inclusion probabilities, $\pi_{ij}=P\left(i\in S_{1st}\&j\in S_{1st}\right)$. Finally, let $\Delta_{ij}=\pi_{ij}-\pi_{i}\pi_{j}$.
- In the ith psu sampled at the first stage, $i\in S_{1st}$, a subsample of the elements, $S_{i}$, of size $m_{i}$ is selected according to a given sampling design $P\left(S_{i}|S_{1st}\right)$ with first-order inclusion probabilities $\pi_{k|i}=P\left(k\in S_{i}|i\in S_{1st}\right)$ and second-order inclusion probabilities $\pi_{k\ell |i}=P\left(k\in S_{i}\&\ell \in S_{i}|i\in S_{1st}\right)$. Subsampling in a given psu is carried out independently of subsampling in any other psu.

A design-unbiased estimator of $t_{y}$ is the Horvitz–Thompson estimator given by

(1) $${\hat{t}}_{y}=\sum_{i\in S_{1st}}\;\pi_{i}^{-1}{\hat{t}}_{i},$$

where ${\hat{t}}_{i}=\sum_{k\in S_{i}}\;y_{ik}/\pi_{k|i}$. The estimator (1) can be written as ${\hat{t}}_{y}=\sum_{k\in \tilde{S}}\;w_{k}y_{k}$, where $w_{k}=\pi_{k}^{-1}$ with $\pi_{k}=\pi_{i}\times \pi_{k|i}$, and $\tilde{S}$ denotes the sample of elements of size $\sum_{i\in S_{1st}}\;m_{i}$.

The design variance of ${\hat{t}}_{y}$, denoted by $V_{p}\left({\hat{t}}_{y}\right)$, can be unbiasedly estimated by

(2) $$\hat{V}\left({\hat{t}}_{y}\right)=\sum_{i\in S_{1st}}\;\sum_{j\in S_{1st}}\;\frac{\Delta_{ij}}{\pi_{ij}}\frac{{\hat{t}}_{i}}{\pi_{i}}\frac{{\hat{t}}_{j}}{\pi_{j}}+\sum_{i\in S_{1st}}\;\pi_{i}^{-1}{\hat{V}}_{i},$$

where

$${\hat{V}}_{i}=\sum_{k\in S_{i}}\;\sum_{\ell \in S_{i}}\;\frac{\Delta_{k\ell |i}}{\pi_{k\ell |i}}\frac{y_{ik}}{\pi_{k|i}}\frac{y_{i\ell }}{\pi_{\ell |i}}$$

and $\Delta_{k\ell |i}=\pi_{k\ell |i}-\pi_{k|i}\pi_{\ell |i}$. That is, $E_{1}E_{2}\left\{\hat{V}\left({\hat{t}}_{y}\right)|S_{1st}\right\}=V_{p}\left({\hat{t}}_{y}\right)$, where $E_{1}(\cdot )$ denotes the expectation with respect to the first-stage sampling design, and $E_{2}\left(\cdot |S_{1st}\right)$ denotes the expectation with respect to the second-stage sampling design conditionally on $S_{1st}$. In the case of simple random sampling without replacement at both stages, the estimator (2) reduces to

(3) $$\hat{V}\left({\hat{t}}_{y,\pi }\right)=N^{2}\left(1-\frac{n}{N}\right)\frac{s_{t}^{2}}{n}+\frac{N}{n}\sum_{i\in S}\;M_{i}^{2}\left(1-\frac{m_{i}}{M_{i}}\right)\frac{s_{yi}^{2}}{m_{i}},$$

where

$$s_{t}^{2}=\frac{1}{n-1}\sum_{i\in S_{1st}}\;{\left({\hat{t}}_{i}-\frac{\sum_{i\in S}\;\;{\hat{t}}_{i}}{n}\right)}^{2}$$

and

$$s_{yi}^{2}=\frac{1}{m_{i}-1}\sum_{k\in S_{i}}\;{\left(y_{ik}-{\overset{‾}{y}}_{i}\right)}^{2}$$

with ${\overset{‾}{y}}_{i}=m_{i}^{-1}\sum_{k\in S_{i}}\;y_{ik}$.

For general two-stage sampling designs, the computation of (2) is cumbersome as it requires the availability of the second-order inclusion probabilities at each stage. A simplified variance estimator is given by

(4) $${\hat{V}}_{sim}\left({\hat{t}}_{y}\right)=\sum_{i\in S_{1st}}\;\sum_{j\in S_{1st}}\;\frac{\Delta_{ij}}{\pi_{ij}}\frac{{\hat{t}}_{i}}{\pi_{i}}\frac{{\hat{t}}_{j}}{\pi_{j}}.$$

That is, only the first term of (2) is kept. The bias of ${\hat{V}}_{\text{sim}\;}\left({\hat{t}}_{y}\right)$, which is always negative, is expected to be small provided that the first-stage sampling fraction, $f_{1}=n/N$, is small; see [12,13].

An alternative simplified variance estimator can be obtained by pretending that the psus are selected with replacement. It is given by

(5) $$\hat{V}\left({\hat{t}}_{y,wr}\right)=\frac{1}{n(n-1)}\sum_{i=1}^{n}\;{\left(\frac{{\hat{t}}_{i}}{p_{i}}-{\hat{t}}_{y,wr}\right)}^{2},$$

where ${\hat{t}}_{y,wr}=\sum_{i\in S}\;\frac{{\hat{t}}_{i}}{np_{i}}$, with $p_{i}$ denoting the probability of selection of the ith psu at any given draw. If the first-stage sampling fraction, $f_{1}$, is small, we expect (5) to suffer from a small positive bias. Unlike (4), the estimator does not require the availability of the second-order inclusion probabilities $\pi_{ij}$.

So far, we have considered the case of a population total $t_{y}$. In practice, it may be of interest to estimate more complex parameters such as distribution functions and quantiles. Let $\theta_{N}$ be defined as the solution of the following census estimating equation:

(6) $$U_{N}(\theta )=\frac{1}{M_{0}}\sum_{i\in U}\;\sum_{k\in U_{i}}\;u\left(y_{ik};\theta \right)=0,$$

where $u\left(y_{ik};\theta \right)$ can be either a smooth (i.e., a function that is differentiable and whose derivatives are continuous) or a non-smooth function of θ. When u(⋅) is smooth, the solution of (6) is called a smooth parameter; otherwise, it is called a non-smooth parameter. Common parameters include: (i) the population mean obtained with $u\left(y_{ik};\theta \right)=y_{ik}-\theta$; (ii) the finite population distribution function obtained with $u\left(y_{ik};\theta \right)=I\left(y_{ik}\leq t\right)-\theta ,t\in R$; (iii) the τ-th population percentile obtained with $u\left(y_{ik};\theta \right)=I\left(y_{ik}\leq \theta \right)-\tau$. The population mean is an example of a smooth parameter, whereas distribution functions and quantiles are examples of non-smooth parameters.

An estimator $\hat{\theta }$ of $\theta_{N}$ can be obtained by solving the following sample estimating equation:

(7) $${\hat{𝒰}}_{S}(\theta )=\frac{1}{M_{0}}\sum_{k\in \tilde{S}}\;w_{k}u\left(y_{k};\theta \right)=0.$$

The variance of $\hat{\theta }$ may be obtained using a first-order Taylor expansion or by using a resampling method such as balanced repeated replication, jackknife, and bootstrap; see [14] for a discussion of resampling methods. In the remainder of this paper, we confine to bootstrap.

## 3. Bootstrap Procedures for Simple Random Sampling without Replacement at Both Stages

In this section, we describe several bootstrap algorithms for a two-stage sampling design with simple random sampling without replacement at both stages.

### 3.1. The Algorithm of Rescaled Bootstrap

Rao and Wu [2] proposed a rescaled bootstrap algorithm for both uni-stage and two-stage sampling designs. Because the rescaling factor is applied to the y-values, this method is applicable to smooth statistics but not to the case of non-smooth statistics such as quantiles. The algorithm can be described as follows:

Step 1. Draw a sample of size n psus from $S_{1st}$, according to simple random sampling with replacement.
Step 2. From each psu selected in Step 1, select a sample of elements, of size $m_{i}$ according to simple random sampling with replacement. For a psu selected more than once in Step 1, perform independent subsampling.
Step 3. Let $y_{ik}^{*}$ be the y-value of the kth bootstrap element in the ith bootstrap psu and $m_{i}^{*}$ be the $m_{i}$-value of the ith bootstrap psu and $M_{i}^{*}$ is defined similarly. Let
$${\tilde{y}}_{ik}=\hat{\overset{‾}{ϒ}}+{\left\{\frac{n\left(1-f_{1}\right)}{n-1}\right\}}^{1/2}\left(\frac{{\overset{ˆ}{t}}_{i}^{*}}{{\overset{‾}{M}}_{0}}-\hat{\overset{‾}{Y}}\right)+{\left\{\frac{m_{i}^{*}f_{1}\left(1-f_{2i}^{*}\right)}{m_{i}^{*}-1}\right\}}^{1/2}\left(\frac{M_{i}^{*}y_{ik}^{*}}{{\overset{‾}{M}}_{0}}-\frac{{\hat{t}}_{i}^{*}}{{\overset{‾}{M}}_{0}}\right),$$
  where $f_{2i}^{*}=m_{i}^{*}/M_{i}^{*},{\hat{t}}_{i}^{*}=\frac{M_{i}^{*}}{m_{i}^{*}}\sum_{k=1}^{m_{i}^{*}}\;y_{ik},{\overset{‾}{M}}_{0}=M_{0}/N$ and $\hat{\overset{‾}{Y}}=\frac{1}{M_{0}}\frac{N}{n}\sum_{i\in S_{1st}}\;\frac{M_{i}}{m_{i}}\sum_{k\in S_{i}}\;y_{ik}$.
Step 4. Compute ${\hat{\theta }}^{*}$ using the same formulae that were used to obtain the original point estimator.
Step 5. Repeat Steps 1–4 a large number of times, B, to obtain ${\hat{\theta }}_{1}^{*},\ldots ,{\hat{\theta }}_{B}^{*}$.
Step 6. The bootstrap variance estimator is ${var}^{*}\left({\hat{\theta }}^{*}\right)$. In practice, the Monte Carlo approximation of ${var}^{*}\left({\hat{\theta }}^{*}\right)$ is applied
$${\hat{var}}^{*}=\frac{1}{B-1}\sum_{b=1}^{B}\;{\left({\hat{\theta }}_{b}^{*}-\overset{-}{\theta^{*}}\right)}^{2},$$
  where $\hat{{\hat{\theta }}^{*}}=B^{-1}\sum_{b=1}^{B}\;{\hat{\theta }}_{b}^{*}$.

Rao and Wu [2] showed that in the case of a population total, the above algorithm matches the standard variance estimator (3). Rao *et al.* [4] proposed a weighted version of the Rao–Wu method, whereby the rescaling is applied to the sampling weights rather than the y-values; see also [10]. The method of Rao *et al.* [4] is described in Section 4.

### 3.2. The Algorithm of Mirror-Match Bootstrap

Sitter [3] proposed an extension of his mirror-match bootstrap to the case of a two-stage sampling design. In [3], the algorithm assumed that the number of repetetions $k_{1}$ and $k_{2i}$ (see below) are integers. It can be described as follows:

Step 1. Choose $1\leq n^{\prime }<n$ and draw a sample of size $n^{\prime }$ psus from $S_{1st}$, according to simple random sampling without replacement.
Step 2. Repeat Step $1k_{1}=n\left(1-f_{1}^{*}\right)/\left\{n^{\prime }\left(1-f_{1}\right)\right\}$ times independently to obtain a bootstrap sample of psus of size $n^{*}=k_{1}n^{\prime }$, where $f_{1}^{*}=n^{\prime }/n$.
Step 3. Choose $1\leq m_{i}^{\prime }<m_{i}$ and draw according to simple random sampling without replacement $m_{i}^{\prime }$ units within the ith psu obtained in Steps 1 and 2.
Step 4. Repeat Step 3 $\;k_{2i}=Nm_{i}\left(1-f_{2i}^{*}\right)/\left\{n^{*}m_{i}^{\prime }\left(1-f_{2i}\right)\right\}$ times independently to obtain a bootstrap sample of size $m_{i}^{*}=k_{2i}m_{i}^{\prime }$ from the ith psu drawn in Step 3, where $f_{2i}^{*}=m_{i}^{\prime }/m_{i}$.
Step 5. Compute ${\hat{\theta }}^{*}$ using the same formulae that were used to obtain the original point estimator.
Step 6. Repeat Steps 1–5 a large number of times, B, to obtain ${\hat{\theta }}_{1}^{*},\ldots ,{\hat{\theta }}_{B}^{*}$.
Step 7. The bootstrap variance estimator is ${var}^{*}\left({\hat{\theta }}^{*}\right)$. In practice, the Monte Carlo approximation of ${var}^{*}\left({\hat{\theta }}^{*}\right)$ is applied
$${\hat{var}}^{*}=\frac{1}{B-1}\sum_{b=1}^{B}\;{\left({\hat{\theta }}_{b}^{*}-\overset{-}{{\hat{\theta }}^{*}}\right)}^{2},$$
  where $\overset{-}{{\hat{\theta }}^{*}}=B^{-1}\sum_{b=1}^{B}\;{\hat{\theta }}_{b}^{*}$.

Sitter [3] showed that in the case of a population total, the above algorithm matches the standard variance estimator (3). If $k_{1}$ and $k_{2i}$ are not integers, a randomization between bracketing integer values is proposed in [15].

### 3.3. The Algorithm of Pseudo-Population Bootstrap

Sitter [16] proposed a pseudo-population bootstrap algorithm, referred to as the without-replacement bootstrap (BWO) method, in the case of uni-stage and two-stage sampling designs. We focus on the latter. In [16], the algorithm assumed that the quantities $k_{1},n^{\prime },k_{2i}$, and $m_{i}^{\prime }$ (see below) are integers. It can be described as follows:

Step 1: Create a pseudo-population by replicating each psu in $S_{1st}k_{1}$ times and each unit within the ith psu $k_{2i}$ times. Let ${𝒰}^{\prime }$ be the resulting pseudo-population consisting of $N^{\prime }=nk_{1}$ psus, ${𝒰}_{1}^{\prime },\ldots ,{𝒰}_{N^{\prime }}^{\prime }$, of size $M_{1}^{\prime },\ldots ,M_{N^{\prime }}^{\prime }$, where there exists $j\in S_{1st}$ such that $M_{i}^{\prime }=m_{j}k_{2j}$. Let $M_{0}^{\prime }=\sum_{i\in {𝒰}^{\prime }}\;M_{i}^{\prime }$ be the total number of elements in the pseudo-population.
Step 2: From the pseudo-population ${𝒰}^{\prime }$, select a sample of psus, $S_{1st}^{*}$, of size $n^{\prime }$, according to simple random sampling without replacement. In each selected psu, select a sample, $S_{i}^{*}$, of size $m_{i}^{\prime }$ according to simple random sampling without replacement.
Step 3: Compute ${\hat{\theta }}^{*}$ using the formulae that were used to obtain the original point estimator.
Step 4: Repeat Steps 2 and 3 a large number of times, B, to obtain ${\hat{\theta }}_{1}^{*},\ldots ,{\hat{\theta }}_{B}^{*}$.
Step 5: The bootstrap variance estimator is ${var}^{*}\left({\hat{\theta }}^{*}\right)$. In practice, the Monte Carlo approximation of ${var}^{*}\left({\hat{\theta }}^{*}\right)$ is applied
$${\hat{var}}^{*}=\frac{1}{B-1}\sum_{b=1}^{B}\;{\left({\hat{\theta }}_{b}^{*}-\overset{-}{{\hat{\theta }}^{*}}\right)}^{2},$$
  where $\overset{-}{{\hat{\theta }}^{*}}=B^{-1}\sum_{b=1}^{B}\;{\hat{\theta }}_{b}^{*}$.

In the case of the population total (or the population mean), Sitter [16] showed that the bootstrap variance estimator reduces to the standard variance estimator provided that $n^{\prime }$ and $k_{1}$ satisfy

(8) $$f_{1}^{\prime }=f_{1}\text{ and }\frac{k_{1}(n-1)}{n^{\prime }\left(k_{1}n-1\right)}=\frac{1}{n},$$

and $m_{i}^{\prime }$ and $k_{2i}$ satisfy

(9) $$f_{2i}^{\prime }=f_{2i}\text{ and }\frac{k_{2i}\left(m_{i}-1\right)}{n^{\prime }\left(k_{2i}m_{i}-1\right)}=\frac{f_{1}}{nm_{i}},$$

where $f_{1}^{\prime }=n^{\prime }/N^{\prime }$ and $f_{2i}^{\prime }=m_{i}^{\prime }/M_{i}^{\prime }$, for each i. If $k_{1},n^{\prime },k_{2i}$, and $m_{i}^{\prime }$ are not integers, a randomization between bracketing integer values was proposed in [15]. In Appendix A, we show that, if we define $k_{1},n^{\prime },k_{2i}$, and $m_{i}^{\prime }$ as in (8) and (9), the bootstrap variance estimator does not reduce to the standard variance estimator in (3). We suggest a modification to (8) and (9) so that the bootstrap variance estimators reduces to the standard variance estimator (3). In the simulation study (see Section 5), we show that the modified version of Sitter [16] works well in terms of bias and coverage probability of confidence intervals.

### 3.4. The Algorithm of Bernoulli Bootstrap

Funaoka *et al.* [17] proposed two bootstrap procedures, referred to as Bernoulli bootstrap, for stratified three-stage sampling. Here, we consider the special case of two-stage sampling. Funaoka *et al.* [17] proposed a short-cut algorithm and a general algorithm. The general algorithm can handle any combination of sample sizes but is more computationally intensive. The general algorithm can be described as follows:

Step 1. Draw a sample, ${\tilde{S}}_{1}$, of size $n^{\prime }=n-1$, from the original sample of clusters, $S_{1st}$, according to simple random sampling with replacement. Generate n Bernoulli random variables, $I_{1i}$, with probability
$$p_{1}=1-\frac{1-f_{1}}{2\left(1-n^{-1}\right)}.$$
  For each $i\in S_{1st}$, keep the ith cluster in the bootstrap sample $S^{*}$ and go to Step 2, if $I_{1i}=1$, and replace the ith cluster with one randomly selected cluster from ${\tilde{S}}_{1st}$, if $I_{1i}=0$.
Step 2. For cluster i kept in Step 1, draw a sample, ${\tilde{S}}_{2i}$, of size $m_{i}^{\prime }=m_{i}-1$, from the original sample $S_{2i}$ according to simple random sampling with replacement. Generate $m_{i}$ Bernoulli random variable, $I_{2ij}$, with probability
$$p_{2i}=1-\frac{f_{1}\left(1-f_{2i}\right)}{2p_{1}^{-1}\left(1-m_{i}^{-1}\right)}.$$
  For each $(ij)\in S_{2i}$, keep the (ij)th element in the bootstrap sample, $S^{*}$, if $I_{2ij}=1$, and replace it with one randomly selected element from ${\tilde{S}}_{2i}$, if $I_{2ij}=0$.
Step 3. Compute ${\hat{\theta }}^{*}$ using the formulae that were used to obtain the original point estimator.
Step 4. Repeat Steps 1–3 a large number of times, B, to obtain ${\hat{\theta }}_{1}^{*},\ldots ,{\hat{\theta }}_{B}^{*}$.
Step 5. The bootstrap variance estimator is ${var}^{*}\left({\hat{\theta }}^{*}\right)$. In practice, the Monte Carlo approximation of ${var}^{*}\left({\hat{\theta }}^{*}\right)$ is applied
$${\hat{var}}^{*}=\frac{1}{B-1}\sum_{b=1}^{B}\;{\left({\hat{\theta }}_{b}^{*}-\overset{-}{{\hat{\theta }}^{*}}\right)}^{2},$$
  where $\overset{-}{{\overset{ˆ}{\theta }}^{*}}=B^{-1}\sum_{b=1}^{B}\;{\hat{\theta }}_{b}^{*}$.

Funaoka *et al.* [17] argued that the resulting bootstrap variance estimator is consistent for both smooth and non-smooth parameters.

### 3.5. The Algorithm of Bootstrap Weight Approach

Preston [18] proposed a bootstrap weight approach for stratified three-stage sampling. Here, we focus on the special case of two-stage sampling. The algorithm can be described as follows:

Step 1. Draw a sample of size $n^{\prime }$ psus from $S_{1st}$, according to simple random sampling without replacement. Let $\delta_{i}=1$ if the ith psu is selected and $\delta_{i}=0$, otherwise.
Step 2. Define the psu bootstrap weights:
$$w_{i}^{*}=\left\{1+\lambda_{1}\left(\frac{n}{n^{\prime }}\delta_{i}-1\right)\right\}\pi_{i}^{-1},$$
  where $\lambda_{1}={\left\{n^{\prime }\left(1-f_{1}\right)/\left(n-n^{\prime }\right)\right\}}^{1/2}$.
Step 3. Within each of the sample of psus selected in Step 1, draw a simple random sample without replacement, of size $m_{i}^{\prime }$. Let $\delta_{ik}=1$ if the kth element in the ith psu is selected and $\delta_{ik}=0$, otherwise. We define the conditional element bootstrap weights:
$$w_{ik}^{*}=\left\{1+\lambda_{1}\left(\frac{n}{n^{\prime }}\delta_{i}-1\right)+\lambda_{2i}{\left(\frac{n}{n^{\prime }}\right)}^{1/2}\delta_{i}\left(\frac{m_{i}}{m_{i}^{\prime }}\delta_{ik}-1\right)\right\}\frac{\pi_{i}^{-1}}{w_{i}^{*}}\pi_{k|i}^{-1},$$
  where $\lambda_{2i}={\left\{m_{i}^{\prime }f_{1}\left(1-f_{2i}\right)/\left(m_{i}-m_{i}^{\prime }\right)\right\}}^{1/2}$.
Step 4. Compute ${\hat{\theta }}^{*}$ using the formulae that were used to obtain the original point estimator with the original weights replaced by the bootstrap weights $w_{ik}^{*}$.
Step 5. Repeat Steps 1–4 a large number of times, B, to obtain ${\hat{\theta }}_{1}^{*},\ldots ,{\hat{\theta }}_{B}^{*}$.
Step 6. The bootstrap variance estimator is ${var}^{*}\left({\hat{\theta }}^{*}\right)$. In practice, the Monte Carlo approximation of ${var}^{*}\left({\hat{\theta }}^{*}\right)$ is applied
$${\hat{var}}^{*}=\frac{1}{B-1}\sum_{b=1}^{B}\;{\left({\hat{\theta }}_{b}^{*}-\overset{-}{{\hat{\theta }}^{*}}\right)}^{2},$$
  where $\overset{-}{{\hat{\theta }}^{*}}=B^{-1}\sum_{b=1}^{B}\;{\hat{\theta }}_{b}^{*}$.

In the case of the population total, Preston [18] showed that the bootstrap variance estimator reduces to the textbook variance estimator (3). He suggested that the choice of $n^{\prime }=\lfloor n/2\rfloor$ and $m_{i}^{\prime }=\left\lfloor m_{i}/2\right\rfloor$ will be optimal and lead to non-negative bootstrap weights, where ⌊⋅⌋ denotes the integer part.

## 4. Bootstrap Procedures for Unequal Probability Sampling Designs

### 4.1. The Procedure of Bootstrap Weight Approach

Rao *et al.* [4] proposed a bootstrap weight approach for stratified multi-stage sampling designs. Unlike the method of Rao and Wu [2], it can be applied to estimate the variance of smooth and non-smooth parameters (e.g., quantiles).

Step 1. Select $n^{\prime }$ psus according to simple random sampling with replacement from $S_{1st}$.
Step 2. Define the bootstrap weight as
$$w_{ik}^{*}=\left\{1+{\left(\frac{n^{\prime }}{n-1}\right)}^{1/2}\left(\frac{nn_{i}^{*}}{n^{\prime }}-1\right)\right\}\pi_{i}^{-1}\pi_{k|i}^{-1},$$
  where $n_{i}^{*}$ denotes the number of times the ith psu is selected in the bootstrap sample.
Step 3. Compute ${\hat{\theta }}^{*}$ using the formulae that were used to obtain the original point estimator with the original weights replaced by the bootstrap weights $w_{ik}^{*}$.
Step 4. Repeat Steps 1–3  B times to obtain ${\hat{\theta }}_{1}^{*},\ldots ,{\hat{\theta }}_{B}^{*}$.
Step 5. The bootstrap variance estimator is ${var}^{*}\left({\hat{\theta }}^{*}\right)$. In practice, the Monte Carlo approximation of ${var}^{*}\left({\hat{\theta }}^{*}\right)$ is applied
$${\hat{var}}^{*}=\frac{1}{B-1}\sum_{b=1}^{B}\;{\left({\hat{\theta }}_{b}^{*}-\overset{-}{{\hat{\theta }}^{*}}\right)}^{2},$$
  where $\overset{-}{{\hat{\theta }}^{*}}=B^{-1}\sum_{b=1}^{B}\;{\hat{\theta }}_{b}^{*}$.

The algorithm of Rao *et al.* [4] leads to consistent variance estimators provided that the first-stage sampling fraction is negligible. The choice $0<n^{\prime }\leq n-1$ leads to non-negative bootstrap weights.

### 4.2. The Procedure of Pseudo-Population Bootstrap Approach

Chauvet [8] proposed a pseudo-population bootstrap approach (PPB) in the case of unequal two-stage sampling designs. It can be described as follows:

Step 1. Each unit $k\in S_{i}$ is duplicated $\left[\pi_{k|i}^{-1}\right]$ times to create a second-stage pseudo-population denoted by ${𝒰}_{i}^{*},i\in S_{1st}$, where [⋅] denotes the closet integer.
Step 2. Each pair $\left(S_{i},{𝒰}_{i}^{*}\right)$ is duplicated $\left\lfloor \pi_{i}^{-1}\right\rfloor$ times. The population of pairs is completed by selecting a sample in the set $\left\{\left(S_{i},{𝒰}_{i}^{*}\right);i\in S_{1st}\right\}$ by means of sampling design with first-order inclusion probabilities $\pi_{i}^{-1}-\left\lfloor \pi_{i}^{-1}\right\rfloor$. This leads to the pseudo-population ${𝒰}^{*}$.
Step 3. Select a first-stage bootstrap sample $S_{1st}^{*}$ from ${𝒰}^{*}$ using the original first-stage sampling design with first-order inclusion probabilities $\pi_{i}$.
Step 4. Select a second-stage bootstrap sample $S_{i}^{**}$ from ${𝒰}_{i}^{*}$ using the original second-stage sampling design. We set $S_{i}^{*}=S_{i}^{**}$ with probability $\pi_{i}$ and $S_{i}^{*}=S_{i}$ with probability $1-\pi_{i}$. This procedure is applied to each pair $\left(S_{i},U_{i}^{*}\right)\in S_{1st}^{*}$. The union of the $S_{i}^{*}$’s leads to the bootstrap sample $S^{*}$.
Step 5. Compute ${\hat{\theta }}^{*}$ using the formulae that were used to obtain the original point estimator.
Step 6. Steps 3–5 are repeated $B_{PPB}$ times to obtain the bootstrap statistics ${\hat{\theta }}_{1}^{*},\ldots ,{\hat{\theta }}_{PPB}^{*}$. Let
$${\hat{v}}^{*}=\frac{1}{B_{PPB}-1}\sum_{b=1}^{B_{PPB}}\;{\left({\hat{\theta }}_{b}^{*}-\overset{-}{{\overset{ˆ}{\theta }}^{*}}\right)}^{2},$$
  where $\overset{-}{{\hat{\theta }}^{*}}=B_{PPB}^{-1}\sum_{b=1}^{B_{PPB}}\;{\hat{\theta }}_{b}^{*}$.
Step 7. Steps 2–6 are repeated $A_{PPB}$ times to obtain ${\hat{v}}_{1}^{*},\ldots ,{\hat{v}}_{A_{PPB}}^{*}$. The variance of $\hat{\theta }$ is estimated by
$${\hat{var}}^{*}=\frac{1}{A_{PPB}}\sum_{a=1}^{A_{PPB}}\;{\hat{v}}_{a}^{*}$$

Chauvet [8] showed that in the case of high entropy sampling design (e.g., [19–22]) at both stages, the above algorithm leads to a consistent estimator in the context of a population total. In the case of a fixed-size sampling design, Chauvet [8] suggested completing the pseudo-population in Step 2, by applying Poisson sampling design with inclusion probabilities $\pi_{i}^{-1}-\left\lfloor \pi_{i}^{-1}\right\rfloor$.

## 5. Simulation Study

We conducted a simulation study to assess the performance of the bootstrap procedures described in Sections 3 and 4 in terms of bias, stability, and coverage rate of confidence intervals based on the t-distribution with n-1 degrees of freedom. The simulation study was carried out using the R software. We created two finite populations consisting of N=200 primary sampling units. The cluster sizes $M_{i}$ were generated according to a Poisson distribution with a mean equal to 50. In each population, we generated a survey variable y according to

$$y_{ij}=10+x_{i}+\varepsilon_{ij},$$

where

(10) $$x_{i}\sim N\left(0,\sqrt{\frac{\rho }{1-\rho }}\right)\text{ and }\varepsilon_{ij}\sim N\left(M_{i},1\right).$$

The parameter ρ in (10) was set to 0.1 for Population 1 and 0.3 for Population 2. We were interested in estimating the population total of the y-variable, $t_{y}$, as well as the finite population median.

From each population, we selected K=3000 samples according to a two-stage sampling design:

- At the first stage, we selected n psus according to two sampling designs: simple random sampling without replacement and inclusion probability-proportional-to-size randomized systematic sampling. The value of n was set to n=10 which corresponds to a first-stage sampling fraction, $f_{1}=5\%$, and n=40, which corresponds to $f_{1}=20\%$.
- At the second stage, $m_{i}=5$ elements within each psu selected at the first stage were selected according to simple random sampling without replacement.

In each sample, we computed the estimator ${\hat{t}}_{y}$ given by (1). Its variance was estimated using the variance estimation procedures listed in Table 1. Except of the procedure of Chauvet [8], we used B=500 bootstrap samples for all the other bootstrap procedures. For the procedure of Chauvet [8], we used $A_{PPB}=10$ and $B_{PPB}=50\left(B=A_{PPB}\times B_{PPB}\right)$.

As a measure of bias of a variance estimator $\hat{var}$, we computed its Monte Carlo percent relative bias

$$RB_{MC}\left(\hat{var}\right)=100\times \frac{E_{MC}\left(\hat{var}\right)-V_{MC}\left({\hat{t}}_{y}\right)}{V_{MC}\left({\hat{t}}_{y}\right)},$$

where $E_{MC}(\hat{var})$ denotes the Monte Carlo expectation of $\hat{var}$ and $V_{MC}\left({\hat{t}}_{y}\right)$ denotes the Monte Carlo variance estimator of ${\hat{t}}_{y}$. As a measure of stability of a variance estimator $\hat{var}$, we computed its Monte Carlo percent coefficient of variation given by

$$CV_{MC}=100\times \frac{\sqrt{V_{MC}(\hat{var})}}{E_{MC}(\hat{var})},$$

where $V_{MC}(\hat{var})$ denotes the Monte Carlo variance estimator of $\hat{var}$. Finally, we computed the Monte Carlo coverage rate of t-based confidence intervals and their corresponding Monte Carlo average length.

Tables 2–5 show the results for the bootstrap methods in the case of SRSWOR/SRSWOR. Table 2 shows the Monte Carlo percent relative bias of the bootstrap variance estimators. For the population total, all the procedures led to small biases for $f_{1}=5\%$ with the value of absolute relative bias ranging from 1.08% to 8.66%. For the population median, except for the method of Rao and Wu [2], the other procedures led to good results with an absolute relative bias ranging from 0.02% to 16.58%. As expected, the method of Rao and Wu [2] led to substantial bias because it cannot be applied to non-smooth statistics. For $f_{1}=20\%$, the absolute relative bias varied between 2.49% and 10.00% for all bootstrap methods except for the method of Rao *et al.* [4] who suffered from a significant positive bias. This can be explained by the fact that the sampling fraction was not small. For $f_{1}=20\%$ the absolute relative bias varied between 2.60% and 13.20% for all bootstrap methods except for the methods of Rao and Wu [2] and Rao *et al.* [4].

Table 3 shows the percent CV. All the bootstrap methods led to similar Monte Carlo coefficients of variation (CV). For $f_{1}=5\%$, the CV varied between 41.2% and 46.0% for the population total, and between 59.0% and 64.3% (except for the method of Rao and Wu [2] that led to a CV of 69.1% for ρ=0.3) for the population median. For $f_{1}=20\%$, the CV varied between 18.9% and 21.0% for the population total, and between 36.4% and 42.3% for the population median.

Tables 4 and 5 show the coverage probability and the average length of 95% confidence intervals based on the t-distribution, respectively. All the bootstrap methods led to good coverage and similar average length except the method of Rao and Wu [2] for the population median. The coverage rate in all cases, except the method of Rao and Wu [2] for the population median, varied between 93.17% and 96.73%.

Tables 6–9 show the results for the bootstrap methods in the case of randomized IPPS systematic/SRSWOR. Table 6 shows the percent relative bias of the bootstrap variance estimators. The method of Chauvet [8] worked generally better than the method of Rao *et al.* [4] in terms of relative bias, especially in the case of the population median. The percent CVs presented in Table 7 were very similar for both methods.

Tables 8 and 9 respectively show the coverage probability and the average length of the 95 percent confidence intervals based on the t-distribution for both methods. Both bootstrap methods led to good coverage and similar average length. The coverage rate in all cases varied from 93.23% to 96.43%.

## 6. Final Remarks

The results of the simulation studies suggest that most bootstrap procedures work well in terms of bias and coverage rate of confidence intervals for estimating smooth or non-smooth parameters. An exception is the method of Rao and Wu [2] for quantiles and the method of Rao *et al.* [4] for appreciable first-stage sampling fractions. In terms of stability of the variance estimators, there is little difference between the bootstrap procedures. Our results are aligned with those of Saigo [23] who empirically compared four bootstrap procedures in the context of stratified three-stage sampling with simple random sampling without replacement at each stage: the procedure of Funaoka *et al.* [17], the mirror match bootstrap of Sitter [3], the method of Rao *et al.* [4], and the BWO method of Sitter [16].

In this paper, we have compared several bootstrap algorithms in the context of a two-stage sampling design. The algorithms were described in an ideal setup. In practice, the design weights $\pi_{k}^{-1}$ undergo a weighting process that involves a nonresponse adjustment followed by some form of calibration, whose goal is to ensure consistency between survey estimates and known population quantities. When the first-stage sampling fraction is small, the method of Rao *et al.* [4] is typically used in surveys conducted by national statistical offices. To account for unit nonresponse and calibration, the bootstrap weights need to undergo the same weighting process (non-response adjustment and calibration) that was used in the original sample were.

Bootstrap may be used to estimate the variance of imputed estimators in the context of imputation for item nonresponse. If the first-stage sampling fraction is small, one can reimpute the missing values in each bootstrap sample using the same imputation procedure that was utilized in the original sample, see [24]. The case of non-negligible first-stage sampling fractions requires further research.

We end this paper by pointing out a very recent paper by Beaumont and Émond [25], who proposed a bootstrap weight approach for multi-stage sampling designs. It would be interesting to compare this method to the procedures considered in this paper.

## Figures and Tables

**Table 1.** Variance estimation procedures for each population parameter/sampling design.

Sampling Design	Population Total	Population Median
SRSWOR/SRSWOR	Textbook variance estimator	Linearization variance estimator
	Rao and Wu [2]	Rao and Wu [2]
	Rao *et al.* [4]	Rao *et al.* [4]
	Modified Sitter	Modified Sitter
	Funaoka *et al.* [17]	Funaoka *et al.* [17]
	Chauvet [8]	Chauvet [8]
	Preston [18]	Preston [18]
IPPSWOR/SRSWOR	Textbook variance estimator	Linearization variance estimator
	Rao *et al.* [4]	Rao *et al.* [4]
	Chauvet [8]	Chauvet [8]

**Table 2.** Monte Carlo percent RB of the bootstrap variance estimators to estimate the variance of the point estimator based on 3000 samples selected according to SRSWOR/SRSWOR.

		ρ=0.1		ρ=0.3	
		$f_{1}=5\%$	$f_{1}=20\%$	$f_{1}=5\%$	$f_{1}=20\%$
$\hat{\theta }$	Bootstrap Method	(n=10)	(n=40)	(n=10)	(n=40)
${\hat{t}}_{y}$	Textbook	1.99	5.10	1.28	5.05
	Chauvet [8]	−7.79	2.49	−8.39	2.54
	Rao *et al.* [4]	6.99	29.07	6.27	29.38
	Rao and Wu [2]	8.66	10.00	6.96	9.20
	Preston [18]	1.88	5.20	1.13	5.11
	Modified Sitter	6.97	9.93	5.84	9.49
	Funaoka *et al.* [17]	1.80	4.00	1.08	4.15
${\hat{\theta }}_{1/2}$	Textbook	11.71	7.42	19.31	10.96
	Chauvet [8]	−0.02	7.56	0.03	8.67
	Rao *et al.* [4]	11.19	20.39	13.05	27.92
	Rao and Wu [2]	930.28	729.81	502.50	367.14
	Preston [18]	10.97	7.58	16.58	11.00
	Modified Sitter	11.81	12.98	10.74	13.20
	Funaoka *et al.* [17]	7.38	2.60	9.21	6.76

**Table 3.** Monte Carlo percent CV based on 3000 samples selected according to SRSWOR/SRSWOR.

		ρ=0.1		ρ=0.3	
		$f_{1}=5\%$	$f_{1}=20\%$	$f_{1}=5\%$	$f_{1}=20\%$
$\hat{\theta }$	Bootstrap Method	(n=10)	(n=40)	(n=10)	(n=40)
${\hat{t}}_{y}$	Textbook	43.4	18.9	45.3	19.8
	Chauvet [8]	43.9	19.9	45.9	20.9
	Rao *et al.* [4]	44.0	20.1	45.8	21.0
	Rao and Wu [2]	41.2	19.3	43.3	20.2
	Preston [18]	43.8	20.2	45.6	21.0
	Modified Sitter	43.6	19.9	45.0	20.6
	Funaoka *et al.* [17]	44.1	20.2	46.0	21.0
${\hat{\theta }}_{1/2}$	Textbook	62.0	37.2	62.4	36.4
	Chauvet [8]	61.0	41.1	60.5	38.9
	Rao *et al.* [4]	59.9	41.1	59.3	38.0
	Rao and Wu [2]	64.3	37.4	69.1	39.7
	Preston [18]	63.8	38.0	64.0	37.3
	Modified Sitter	59.4	40.4	59.0	38.3
	Funaoka *et al.* [17]	59.8	42.3	59.3	39.5

**Table 4.** Coverage rate (CR) of the 95%  t-distribution based confidence intervals constructed using bootstrap standard error estimators based on 3000 samples selected according to SRSWOR/SRSWOR.

		ρ=0.1		ρ=0.3	
		$f_{1}=5\%$	$f_{1}=20\%$	$f_{1}=5\%$	$f_{1}=20\%$
$\hat{\theta }$	Bootstrap Method	(n=10)	(n=40)	(n=10)	(n=40)
${\hat{t}}_{y}$	Textbook	95.33	95.07	95.40	94.73
	Chauvet [8]	94.50	94.87	94.70	94.67
	Rao *et al.* [4]	95.50	96.63	95.77	96.73
	Rao and Wu [2]	96.10	95.33	96.00	95.47
	Preston [18]	95.37	95.07	95.30	94.77
	Modified Sitter	95.80	95.47	95.73	95.30
	Funaoka *et al.* [17]	95.27	94.97	95.33	94.73
${\hat{\theta }}_{1/2}$	Textbook	94.63	95.23	94.27	95.20
	Chauvet [8]	93.90	94.67	93.17	94.57
	Rao *et al.* [4]	95.10	95.97	94.70	96.03
	Rao and Wu [2]	100.00	99.97	99.97	99.93
	Preston [18]	94.50	95.23	93.70	94.80
	Modified Sitter	95.07	95.13	94.67	94.93
	Funaoka *et al.* [17]	94.73	94.27	94.20	94.13

**Table 5.** Average length (AL) of the 95% t-distribution based confidence intervals constructed using bootstrap standard error estimators based on 3000 samples selected according to SRSWOR/SRSWOR.

		ρ=0.1		ρ=0.3	
		$f_{1}=5\%$	$f_{1}=20\%$	$f_{1}=5\%$	$f_{1}=20\%$
$\hat{\theta }$	Bootstrap Method	(n=10)	(n=40)	(n=10)	(n=40)
${\hat{t}}_{y}$	Textbook	21,470.7	8725.5	23,157.5	9721.9
	Chauvet [8]	20,404.4	8612.6	22,012.1	9599.7
	Rao *et al.* [4]	21,978.5	9663.6	23,707.2	10,782.5
	Rao and Wu [2]	22,218.5	8925.4	23,857.5	9910.3
	Preston [18]	21,450.9	8724.4	23,134.4	9718.9
	Modified Sitter	21,988.4	8919.4	23,684.7	9921.4
	Funaoka *et al.* [17]	21,435.4	8674.5	23,119.5	9674.2
${\hat{\theta }}_{1/2}$	Textbook	0.9796	0.4081	1.3847	0.5705
	Chauvet [8]	0.9284	0.4071	1.2717	0.5634
	Rao *et al.* [4]	0.9798	0.4306	1.3537	0.6117
	Rao and Wu [2]	2.9770	1.1343	3.0965	1.1681
	Preston [18]	0.9736	0.4081	1.3654	0.5701
	Modified Sitter	0.9795	0.4175	1.3407	0.5753
	Funaoka *et al.* [17]	0.9634	0.3972	1.3306	0.5582

**Table 6.** Monte Carlo percent RB of the bootstrap variance estimators to estimate the variance of the point estimator based on 3000 samples selected according to IPPS/SRSWOR.

		ρ=0.1		ρ=0.3	
		$f_{1}=5\%$	$f_{1}=20\%$	$f_{1}=5\%$	$f_{1}=20\%$
$\hat{\theta }$	Bootstrap Method	(n=10)	(n=40)	(n=10)	(n=40)
${\hat{t}}_{y}$	Textbook	−1.26	−0.36	−1.98	−0.05
	Chauvet [8]	−13.65	−5.74	−11.86	−3.83
	Rao *et al.* [4]	1.24	9.93	2.06	18.03
${\hat{\theta }}_{1/2}$	Textbook	18.37	3.92	22.58	5.14
	Chauvet [8]	0.16	−3.24	2.65	2.09
	Rao *et al.* [4]	16.45	15.71	17.72	21.56

**Table 7.** Monte Carlo percent CV based on 3000 samples selected according to IPPS/SRSWOR.

		ρ=0.1		ρ=0.3	
		$f_{1}=5\%$	$f_{1}=20\%$	$f_{1}=5\%$	$f_{1}=20\%$
$\hat{\theta }$	Bootstrap Method	(n=10)	(n=40)	(n=10)	(n=40)
${\hat{t}}_{y}$	Textbook	44.9	18.9	44.6	19.0
	Chauvet [8]	46.2	21.0	46.7	21.3
	Rao *et al.* [4]	46.9	22.4	46.1	21.3
${\hat{\theta }}_{1/2}$	Textbook	62.0	37.7	59.3	36.1
	Chauvet [8]	61.3	40.5	61.0	40.1
	Rao *et al.* [4]	60.3	41.2	58.4	37.5

**Table 8.** Coverage rate (CR) of the 95%  t-distribution based confidence intervals constructed using bootstrap standard error estimators based on 3000 samples selected according to IPPS/SRSWOR.

		ρ=0.1		ρ=0.3	
		$f_{1}=5\%$	$f_{1}=20\%$	$f_{1}=5\%$	$f_{1}=20\%$
$\hat{\theta }$	Bootstrap Method	(n=10)	(n=40)	(n=10)	(n=40)
${\hat{t}}_{y}$	Textbook	95.27	95.50	94.73	94.83
	Chauvet [8]	93.23	94.83	93.63	94.70
	Rao *et al.* [4]	95.40	96.07	95.03	96.43
${\hat{\theta }}_{1/2}$	Textbook	94.53	94.63	95.70	93.63
	Chauvet [8]	93.77	93.80	93.27	93.93
	Rao *et al.* [4]	95.17	94.93	95.27	95.43

**Table 9.** Average length (AL) of the 95%  t-distribution based confidence intervals constructed using bootstrap standard error estimators based on 3000 samples selected according to IPPS/SRSWOR.

		ρ=0.1		ρ=0.3	
		$f_{1}=5\%$	$f_{1}=20\%$	$f_{1}=5\%$	$f_{1}=20\%$
$\hat{\theta }$	Bootstrap Method	(n=10)	(n=40)	(n=10)	(n=40)
${\hat{t}}_{y}$	Textbook	7694.4	3401.9	11,021.3	4746.8
	Chauvet [8]	7317.2	3314.3	10,508.3	4650.7
	Rao *et al.* [4]	7772.1	3566.9	11228.1	5152.5
${\hat{\theta }}_{1/2}$	Textbook	0.9797	0.4184	1.3709	0.5597
	Chauvet [8]	0.9247	0.4110	1.2627	0.5472
	Rao *et al.* [4]	0.9736	0.4403	1.3461	0.6011

## Appendix A

In this section, we show that, obtaining $k_{1},n^{\prime },k_{2i}$, and $m_{i}^{\prime }$ from Equations (8) and (9) in the Sitter [16] algorithm in Section 3.3, the resulting bootstrap variance estimator does not reduce to the standard variance estimator in the case of the population total $\theta =t_{y}$. In the case of $\theta =t_{y}$, the bootstrap estimator is

$${\hat{\theta }}^{*}=\frac{N^{\prime }}{n^{\prime }}\sum_{i\in S_{1st}^{*}}\;\frac{M_{i}^{\prime }}{m_{i}^{\prime }}\sum_{k\in S_{i}^{*}}\;y_{ik}^{*}$$

where $y_{ik}^{*}$ is the y-value for the kth selected element in $S_{i}^{*}$ in Step 2. The bootstrap variance of ${\hat{\theta }}^{*}$ is

(A1)
$${\text{var}}^{*}\left({\hat{\theta }}^{*}\right)=V_{1*}E_{2*}\left({\hat{\theta }}^{*}|S_{1st}^{*}\right)+E_{1*}V_{2*}\left({\hat{\theta }}^{*}|S_{1st}^{*}\right),$$

where the subscripts 1* and 2* respectively denote the expectation and the variance with respect to the first-stage and second-stage resampling design in Step 2. Let $t_{i}^{\prime }=\sum_{k\in 𝒰}\;y_{ik}$ be the total of y-values in ${𝒰}_{i}^{\prime }$ in Step 1. The first and the second component of the bootstrap variance estimator ${var}^{*}\left({\hat{\theta }}^{*}\right)$ in (A1) respectively are

$$V_{1*}E_{2*}\left({\hat{\theta }}^{*}|S_{1st}^{*}\right)\;=V_{1*}\left(\frac{N^{\prime }}{n^{\prime }}\sum_{i\in S_{1st}^{*}}\;\;t_{i}^{\prime }\right)\;=\;N^{\prime 2}\frac{1-f_{1}^{\prime }}{n^{\prime }}\frac{1}{N^{\prime }-1}\sum_{i\in {𝒰}^{\prime }}\;\;{\left(t_{i}^{\prime }-\frac{1}{N^{\prime }}\sum_{j\in {𝒰}^{\prime }}\;\;t_{j}^{\prime }\right)}^{2}\;=\;N^{\prime 2}\frac{1-f_{1}^{\prime }}{n^{\prime }}\frac{k_{1}}{k_{1}n-1}\sum_{i\in S_{1st}}\;\;{\left(k_{2i}m_{i}{\overset{‾}{y}}_{i}-\frac{1}{n}\sum_{j\in S_{1st}}\;\;k_{2j}m_{j}{\overset{‾}{y}}_{j}\right)}^{2}$$

and

$$E_{1*}V_{2*}\left({\hat{\theta }}^{*}|S_{1st}^{*}\right)\;=E_{1*}\left\{\frac{N^{\prime 2}}{n^{\prime 2}}\sum_{i\in S_{1st}^{*}}\;\;M_{i}^{\prime 2}\frac{1-f_{2i}^{\prime }}{m_{i}^{\prime }}\frac{1}{M_{i}^{\prime }-1}\sum_{k\in {𝒰}_{i}^{\prime }}\;\;{\left(y_{ik}^{*}-\frac{1}{M_{i}^{\prime }}\sum_{l\in {𝒰}_{i}^{\prime }}\;\;y_{il}^{*}\right)}^{2}\right\}\;\;=\frac{N^{\prime }}{n^{\prime }}\sum_{i\in {𝒰}^{\prime }}\;\;M_{i}^{\prime 2}\frac{1-f_{2i}^{\prime }}{m_{i}^{\prime }}\frac{1}{M_{i}^{\prime }-1}\sum_{k\in {𝒰}_{i}^{\prime }}\;\;{\left(y_{ik}^{*}-\frac{1}{M_{i}^{\prime }}\sum_{l\in {𝒰}_{i}^{\prime }}\;\;y_{il}^{*}\right)}^{2}\;\;=N^{\prime 2}\frac{1}{nn^{\prime }}\sum_{i\in S_{1st}}\;\;{\left(k_{2i}m_{i}\right)}^{2}\frac{1-f_{2i}^{\prime }}{m_{i}^{\prime }}\frac{k_{2i}}{k_{2i}m_{i}-1}\sum_{k\in S_{i}}\;\;{\left(y_{ik}-{\overset{‾}{y}}_{i}\right)}^{2}\;\;=\sum_{i\in S_{1st}}\;\;{\left(N^{\prime }k_{2i}m_{i}\right)}^{2}\frac{1-f_{2i}^{\prime }}{nn^{\prime }m_{i}^{\prime }}\frac{k_{2i}\left(m_{i}-1\right)}{k_{2i}m_{i}-1}s_{yi}^{2},$$

where ${\overset{‾}{y}}_{i}=\sum_{i\in S_{i}}\;/m_{i}$. In [16], it is claimed that the first and the second components of the bootstrap variance estimator respectively are

$$N^{2}\frac{k_{1}(n-1)}{k_{1}n-1}\frac{1-f_{1}^{\prime }}{n^{\prime }}s_{t}^{2},$$

and

$$N^{2}\sum_{i\in S_{1st}}\;M_{i}^{2}\frac{1-f_{2i}^{\prime }}{nn^{\prime }m_{i}^{\prime }}\frac{k_{2i}\left(m_{i}-1\right)}{k_{2i}m_{i}-1}s_{yi}^{2},$$

see Equation (3.5) in Section 3.2 in [16]. This is true only when

(A2)
$$N^{\prime }=k_{1}n=N,\;\text{ and }\;k_{2i}m_{i}=M_{i}\;\text{ for all }i\in S_{1st}.$$

In other words, we have to define $k_{1}=N/n$ and $k_{2i}=M_{i}/m_{i}$ which is contradictory to the way Sitter [16] defined $k_{1},n^{\prime },k_{2i}$, and $m_{i}^{\prime }$ using Equations (8) and (9).

In the following, we suggest how the method of Sitter [16] can be modified. We first define

(A3)
$$k_{1}=\frac{N}{n}\;\text{ and }\;k_{2i}=\frac{M_{i}}{m_{i}}.$$

To have the equality between the first and the second component of the bootstrap variance estimator and the first and the second component of the standard variance estimator in (3), respectively, we need to have

(A4)
$$\frac{k_{1}(n-1)}{k_{1}n-1}\frac{\left(1-f_{1}^{\prime }\right)}{n^{\prime }}=\frac{1-f_{1}}{n}\;⟹\frac{k_{1}(n-1)}{k_{1}n-1}\frac{1-\frac{n^{\prime }}{N}}{n^{\prime }}=\frac{1-f_{1}}{n}\;⟹n^{\prime }=\frac{N}{1+\frac{1-f_{1}}{f_{1}}\frac{k_{1}n-1}{k_{1}(n-1)}}$$

and

(A5)
$$\frac{k_{2i}\left(m_{i}-1\right)}{k_{2i}m_{i}-1}\frac{1-\frac{m_{i}^{\prime }}{M_{i}^{\prime }}}{n^{\prime }m_{i}^{\prime }}=\frac{f_{1}}{nm_{i}}\left(1-f_{2i}\right)\;⟹\frac{1-\frac{m_{i}^{\prime }}{M_{i}}}{m_{i}^{\prime }}=\frac{f_{1}\left(1-f_{2i}\right)}{nm_{i}}\frac{n^{\prime }\left(k_{2i}m_{i}-1\right)}{k_{2i}\left(m_{i}-1\right)}\;⟹m_{i}^{\prime }=\frac{M_{i}}{1+\frac{f_{1}\left(1-f_{2i}\right)}{nf_{2i}}\frac{n^{\prime }\left(k_{2i}m_{i}-1\right)}{k_{2i}\left(m_{i}-1\right)}}$$

Defining $k_{1},n^{\prime },k_{2i}$, and $m_{i}^{\prime }$ as in (A3), (A4) and (A5), the bootstrap variance estimator reduces to the usual variance estimator in (3). In the simulation study, the method “Modified Sitter” refers to the method of Sitter [16] while applying the modified $k_{1},n^{\prime },k_{2i}$, and $m_{i}^{\prime }$ defined in (A3), (A4) and (A5). When $k_{1},n^{\prime },k_{2i}$, or $m_{i}^{\prime }$ is not integer, we simply rounded it to the closest integer value.
